# Assisted Reproductive Technology Surveillance — United States, 2017

**DOI:** 10.15585/mmwr.ss6909a1

**Published:** 2020-12-18

**Authors:** Saswati Sunderam, Dmitry M. Kissin, Yujia Zhang, Amy Jewett, Sheree L. Boulet, Lee Warner, Charlan D. Kroelinger, Wanda D. Barfield

**Affiliations:** ^1^Division of Reproductive Health, National Center for Chronic Disease Prevention and Health Promotion, CDC; ^2^Emory University, School of Medicine, Atlanta, Georgia

## Abstract

**Problem/Condition:**

Since the first U.S. infant conceived with assisted reproductive technology (ART) was born in 1981, both the use of ART and the number of fertility clinics providing ART services have increased steadily in the United States. ART includes fertility treatments in which eggs or embryos are handled in the laboratory (i.e., in vitro fertilization [IVF] and related procedures). Although the majority of infants conceived through ART are singletons, women who undergo ART procedures are more likely than women who conceive naturally to have multiple-birth infants because multiple embryos may be transferred. Multiple births can pose substantial risks for both mothers and infants, including obstetric complications, preterm birth (<37 weeks), and low birthweight (<2,500 g). This report provides state-specific information for the United States (including the District of Columbia and Puerto Rico) on ART procedures performed in 2017 and compares birth outcomes that occurred in 2017 (resulting from ART procedures performed in 2016 and 2017) with outcomes for all infants born in the United States in 2017.

**Period Covered:**

2017.

**Description of System:**

In 1995, CDC began collecting data on ART procedures performed in fertility clinics in the United States as mandated by the Fertility Clinic Success Rate and Certification Act of 1992 (Public Law 102–493 [October 24, 1992]). Data are collected through the National ART Surveillance System (NASS), a web-based data collection system developed by CDC. This report includes data from the 50 states, the District of Columbia, and Puerto Rico.

**Results:**

In 2017, a total of 196,454 ART procedures (range: 162 in Alaska to 24,179 in California) with at least one embryo transferred were performed in 448 U.S. fertility clinics and reported to CDC. These procedures resulted in 68,908 live-birth deliveries (range: 67 in Puerto Rico to 8,852 in California) and 78,052 infants born (range: 85 in Puerto Rico to 9,926 in California). Nationally, the number of ART procedures performed per 1 million women of reproductive age (15–44 years) was 3,040. ART use rates exceeded the national rate in 14 states (Connecticut, Delaware, District of Columbia, Hawaii, Illinois, Maryland, Massachusetts, New Hampshire, New Jersey, New York, Rhode Island, Utah, Vermont, and Virginia). ART use exceeded 1.5 times the national rate in seven states (Connecticut, the District of Columbia, Illinois, Maryland, Massachusetts, New Jersey, and New York).

Nationally, among all ART transfer procedures, the average number of embryos transferred increased slightly with increasing age (1.3 among women aged <35 years, 1.4 among women aged 35–37 years, and 1.5 among women aged >37 years). This year, single-embryo transfer (SET) rates among all embryo-transfer procedures are presented instead of elective single-embryo transfer procedures previously reported. Nationally, SET rates were 67.3% (range: 38.9% in South Dakota to 90.4% in Delaware), 65.0% (range: 23.6% in Puerto Rico to 89.4% in Delaware), and 60.0% (range: 28.6% in Puerto Rico to 83.1% in Delaware) among women aged <35 years, aged 35–37 years, and aged >37 years, respectively.

In 2017, ART contributed to 1.9% of all infants born in the United States (range: 0.4% in Puerto Rico to 5.0% in Massachusetts). Approximately 73.6% of ART-conceived infants were singleton infants. Overall, ART contributed to 14.7% of all multiple births, including 14.7% of all twin infants and 17.3% of all triplets and higher-order infants. ART-conceived twins accounted for approximately 96.5% (18,890 of 19,570) of all ART-conceived infants born in multiple deliveries. The percentage of multiple births was higher among infants conceived with ART (26.4%) than among all infants born in the total birth population (3.4%). Approximately 25.5% of ART-conceived infants were twins, and 0.9% were triplets and higher-order infants.

Nationally, infants conceived with ART contributed to 4.5% of all low birthweight (<2,500 g) infants. Among ART-conceived infants, 20.2% had low birthweight, compared with 8.3% among all infants. ART-conceived infants contributed to 5.3% of all preterm (gestational age <37 weeks) infants. The percentage of preterm births was higher among infants conceived with ART (27.8%) than among all infants born in the total birth population (9.9%).

The percentage of low birthweight among singletons was 8.1% among ART-conceived infants and 6.6% among all infants born. The percentage of preterm births among ART-conceived singleton infants was 14.0%, compared with 8.1% among all singleton infants. The percentages of small for gestational age infants was 7.6% among ART-conceived infants, compared with 9.9% among all infants.

**Interpretation:**

Although singleton infants accounted for the majority of ART-conceived infants, multiple births from ART still contributed to a substantial proportion of all twins, triplets, and higher-order infants born in the United States. Variations in SET rates among states and territories were noted, reflecting variations in embryo-transfer practices among fertility clinics, which might in part account for higher multiple birth from ART observed in some states and territories.

**Public Health Action:**

Reducing the number of embryos transferred and increasing use of SET, when clinically appropriate, can help reduce multiple births and related adverse health consequences for both mothers and infants. Because infants from multiple births are at increased risk for numerous adverse sequelae that cannot be ascertained from the data collected through NASS alone, long-term follow-up for ART infants through integration of existing maternal and infant health surveillance systems and registries with data available from NASS might be useful for monitoring adverse outcomes on a population basis.

## Introduction

Since the birth of the first U.S. infant conceived with assisted reproductive technology (ART) in 1981, use of advanced technologies to overcome infertility has increased, as has the number of fertility clinics providing ART services and procedures in the United States (*1*). In 1992, Congress passed the Fertility Clinic Success Rate and Certification Act of 1992 (Public Law 102–493 [October 24, 1992]), which requires that all U.S. fertility clinics performing ART procedures report data to CDC annually on every ART procedure performed. CDC initiated data collection in 1995 and in 1997 published the first annual ART Fertility Clinic Success Rates Report (*2*). The annual ART Fertility Clinic Success Rates Report presents multiple measures of success for ART, including the percentage of ART procedures and transfers that result in live-birth deliveries.

Although ART has helped millions of women achieve pregnancy, the treatment is associated with potential health risks for both mothers and infants. Because multiple embryos can be transferred in ART procedures, ART might result in multiple-gestation pregnancies and multiple births (*3*–*6*). Risks to the mother from a multiple-birth pregnancy include higher rates of caesarean delivery, maternal hemorrhage, pregnancy-related hypertension, and gestational diabetes (*7*–*10*). Risks to the infant include preterm birth, low birthweight, birth defects, developmental disability, and death (*11*–*14*). In addition, singleton infants conceived with ART might have higher risk for low birthweight and prematurity than singletons not conceived with ART (*15*,*16*). However, recent research suggests that this higher risk might be associated with singleton births resulting from multiple-embryo transfers among patients who were not good candidates for single-embryo transfer (SET) (*17*).

This report was compiled from data provided and verified by ART clinics about ART procedures performed in 2017 and reported to CDC’s Division of Reproductive Health. Data on the use of ART are presented for residents of each U.S. state, the District of Columbia, and Puerto Rico. Data also are reported on outcomes for infants born in 2017 resulting from ART procedures performed in 2016 and 2017. The report examines the proportion of ART among selected outcomes (e.g., multiple births, low birthweight infants, preterm infants, and small for gestational age [SGA] infants) and compares outcomes among ART-conceived infants with outcomes among all infants born in the United States in 2017.

## Methods

### National ART Surveillance System

In 1995, CDC initiated data collection of ART procedures performed in the United States. ART data are obtained from all fertility clinics in the United States that provided and verified information about the outcomes of the ART cycles through the National ART Surveillance System (NASS), a web-based data collection system developed by CDC (https://www.cdc.gov/art/nass/index.html). Clinics that are members of the Society for Assisted Reproductive Technology (SART) can report their data to NASS through SART. Clinics that are not members of SART can enter their data directly into NASS. All clinics must verify the accuracy of the data they reported in the clinic table in the annual ART Fertility Clinic Success Rates Report before finalizing submission to NASS. The data then are compiled by a CDC contractor and reviewed for accuracy. In 2017, 10.0% of clinics did not report their data to CDC and are listed as nonreporting clinics in the 2017 ART Fertility Clinic Success Rates Report, as required by the Fertility Clinic Success Rate and Certification Act of 1992. Because nonreporting clinics tend to be smaller on average than reporting clinics, NASS is estimated to contain information on 98% of all ART procedures in the United States (*1*).

Data collected include patient demographics, medical history, and infertility diagnoses; clinical information pertaining to the ART procedure type; and information regarding resultant pregnancies and births. The data file contains one record per ART procedure (i.e., cycle of treatment performed). Because ART providers typically do not provide continued prenatal care after a pregnancy is established, ART clinics collect information on live births for all procedures from patients and physicians.

### ART Procedures

ART includes fertility treatments in which eggs or embryos are handled in a laboratory (i.e., in vitro fertilization [IVF], gamete intrafallopian transfer, and zygote intrafallopian transfer). More than 99% of ART procedures performed are IVF. Because an ART procedure consists of multiple steps over an interval of approximately 2 weeks, and sometimes longer because of preimplantation genetic testing performed to select euploid embryos or for disease screening, a procedure often is referred to as a cycle of treatment. An ART cycle usually begins with drug-induced ovarian stimulation. If eggs are produced, the cycle progresses to the egg-retrieval stage, which involves surgical removal of the eggs from the ovaries. After the eggs are retrieved, they are combined with sperm in a laboratory during the IVF procedure. For certain IVF procedures (75.0% in 2017) (*1*), a specialized technique (intracytoplasmic sperm injection) is used in which a single sperm is injected directly into the egg. If successful fertilization occurs, the most viable embryos (i.e., those that appear morphologically most likely to develop and implant) are selected for transfer back into the uterus. If an embryo implants in the uterus, a clinical pregnancy is diagnosed by the presence of a gestational sac detectable by ultrasound. On average, less than half of the procedures result in a clinical pregnancy. Most pregnancies will progress to a live-birth delivery, defined as the delivery of one or more live-born infants; however, some result in pregnancy loss (*18*,*19*). ART does not include treatments in which only sperm are handled (i.e., intrauterine insemination) or procedures in which a woman is administered drugs to stimulate egg production without the intention of having eggs retrieved.

ART procedures are classified on the basis of the source of the egg (patient or donor) and the status of the eggs and embryos. Both fresh and thawed embryos can be derived from fresh or frozen eggs of the patient or donor. Patient and donor embryos can be created using sperm from a partner or donor. ART procedures involving fresh eggs and embryos include an egg-retrieval stage. ART procedures that use thawed eggs or embryos do not include egg retrieval because the eggs were retrieved during a previous ART procedure, and either the eggs were frozen or fertilized and the resultant embryos were frozen until the current ART procedure. An ART cycle can be discontinued at any step for medical reasons or by patient choice.

### Birth Data for United States

Data on the total numbers of live births, including singleton and multiple births, in each reporting area in 2017 were obtained from U.S. natality files (*20*–*22*). The natality online databases report counts of live births occurring within the United States to residents and nonresidents. The data are derived from birth certificates.

### Variables and Definitions

Data on ART procedures and birth outcomes are presented by patient’s residence (i.e., state or territory) at the time of treatment, which might not be the same as the location where the procedure was performed. If information on a patient’s residence was missing, residence was assigned as the location where the procedure was performed (0.3% of procedures performed in 2017 and 0.1% of live-birth deliveries occurring in 2017). ART procedures performed in the United States among non-U.S. residents are included in NASS data. However, they are excluded from certain calculations; the appropriate denominators were not available because the women might have delivered outside the United States. To protect confidentiality, table cells with values of 1–4 for ART-conceived infants and 0–9 for all infants are suppressed. The cell suppression criteria for the ART population allows for the representation of some clinics, which carry out only a small number of cycles, while maintaining minimum risks for identification. ART data from U.S. territories (with the exception of Puerto Rico) are not included in this report. In addition, percentages derived from cell values <20 in the denominator have been suppressed because they are unstable.

This report presents data on all procedures initiated with the intent to transfer at least one embryo, including procedures that used thawed embryos for transfer. All cycles in which egg or embryo banking was performed for future ART cycles were excluded. The number of ART procedures performed per 1 million women of reproductive age (15–44 years) was calculated. Data regarding population size were compiled on the basis of July 1, 2017, estimates from the U.S. Census Bureau (*23*). The resulting rate approximates the proportion of women of reproductive age who used ART in each state or territory. This proxy measure of ART use is only an approximation because certain women who use ART fall outside the age range of 15–44 years (approximately 6% of cycles performed in 2017), and certain women might have had more than one procedure during the reporting period.

A live-birth delivery was defined as a birth of one or more infants. A singleton live-birth delivery was defined as a delivery of only one infant who was born live. A multiple live-birth delivery was defined as a delivery of two or more infants, at least one of whom was born live. Low birthweight was defined as <2,500 g, moderately low birthweight as 1,500–2,499 g, and very low birthweight as <1,500 g. Gestational age for births among women who did not undergo ART procedures was calculated using obstetric estimate of gestational age at delivery (*24*). For births to women who underwent fresh ART procedures, gestational age was calculated by subtracting the date of egg retrieval from the birth date and adding 14 days. For births to women who underwent frozen embryo cycles or fresh ART procedures for which the date of retrieval was not available, gestational age was calculated by subtracting the date of embryo transfer from the birth date and adding 17 days (to account for an average of 3 days in embryo culture). Preterm birth was defined as gestational age <37 weeks, late preterm 34–36 weeks, early preterm <34 weeks, and very preterm <32 weeks (*22*).

New in 2017, SET procedures among all embryo-transfer procedures are reported instead of reporting elective SET (eSET) procedures only among patients who used fresh embryos from their own fresh eggs, as in previous reports. In an eSET procedure, only one embryo is selected for transfer from a larger number of available embryos, and the remaining embryos are cryopreserved. In comparison, SET is a procedure in which one embryo is selected for transfer, regardless of how many embryos were available. Therefore, the rate of SET is expected to be higher than the rate of eSET because SET procedures include both eSET procedures and procedures in which only one embryo is available for transfer. This transition from eSET to SET follows changes in clinical practice, such as increasing use of frozen embryos and expanded recommendations to transfer a single embryo regardless of how many embryos are cryopreserved due to increasing use of preimplantation genetic screening, which can facilitate the selection of euploid embryos (*5*,*25*). This guidance adopted a broader approach and recommended single-embryo transfer for patients of any age transferring an euploid embryo, selected with the assistance of preimplantation genetic screening, and for patients aged <38 years with any one of these criteria: 1) availability of quality embryos for cryopreservation, 2) history of live birth after an IVF procedure, 3) availability of vitrified blastocyst stage embryos, or 4) undergoing first frozen-embryo transfer (*5*). The rate of SET was calculated by dividing the total number of SET procedures by the total number of embryo-transfer procedures performed and reported by the following age groups: <35 years, 35–37 years, and >37 years. The average number of embryos transferred by age group (<35 years, 35–37 years, and >37 years) was calculated by dividing the total number of embryos transferred by the total number of embryo-transfer procedures performed among that age group.

The proportion of ART infants among all births in a particular state or territory was used as a second measure of ART use. The proportion of adverse outcomes among ART-conceived infants (e.g., preterm birth) was calculated by dividing the total number of adverse outcomes among ART-conceived infants by the total number of adverse outcomes among all infants born.

The percentage of infants (ART conceived and all infants) born in a state or territory for each plurality group (singleton, multiple, twin, and triplet and higher-order birth) was calculated by dividing the number of infants (ART conceived and all infants) in each plurality group by the total number of infants born (ART conceived and all infants). The percentage of infants with low birthweight and preterm birth was calculated only for singleton births for ART-conceived infants and for all infants by dividing the number of low birthweight or preterm infants among singletons by the total number of singleton infants.

In addition, the percentage of singleton infants who were SGA (defined as <10th percentile of birthweight for gestational age week and limited to 22–44 weeks) was calculated using a reference distribution (*26*). The percentage of singleton SGA infants was calculated for all births by dividing the number of singleton SGA infants in the gestational age category (week) by the total number of singleton infants in that gestational age category for ART-conceived and all infants, respectively.

To assess the proportion of ART births among U.S. births in 2017, ART births were aggregated from two reporting years: 1) infants conceived with ART procedures performed in 2016 and born in 2017 (71.5% of the live-birth deliveries reported to NASS for 2017) and 2) infants conceived with ART procedures performed in 2017 and born in 2017 (28.5% of the live-birth deliveries reported to NASS for 2017).

## Results

### Overview of Fertility Clinics

**FIGURE 1:**
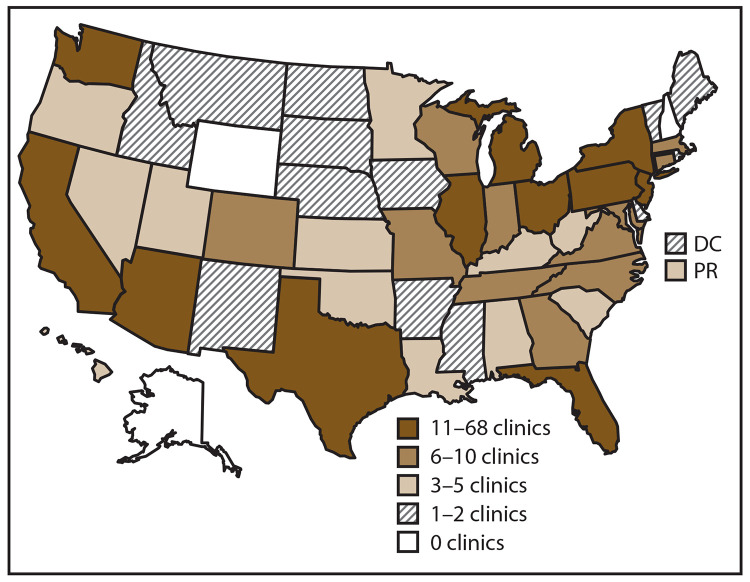
Location and number* of assisted reproductive technology clinics, by quartile — United States and Puerto Rico, 2017 **Abbreviations:** DC = District of Columbia; PR = Puerto Rico. * In 2017, of the 498 clinics in the United States, 448 (90%) submitted data.

In 2017, a total of 498 fertility clinics in the United States performed ART procedures and 448 (90.0%) provided data to CDC, with the majority located in or near major cities (*1*). The number of fertility clinics performing ART procedures varied by state or territory. The states with the largest numbers of fertility clinics providing data were California (68), Texas (41), and New York (40) (Figure 1).

### Number and Type of ART Procedures

The number, type, and outcome of ART procedures performed are provided according to patient’s residence for all 52 states and territories and non-U.S. residents (Table 1). Residency data were missing for approximately 0.3% of procedures performed, and in these cases, the patient’s residence was assigned as the location where the ART procedure was performed. In 2017, approximately 13.8% of ART procedures were performed in a state or territory other than the patient’s state or territory of residence. Non-U.S. residents accounted for approximately 3.6% of ART procedures, 4.3% of ART live-birth deliveries, and 4.5% of ART-conceived infants born.

**TABLE 1:** Number* and outcomes of assisted reproductive technology procedures in which at least one embryo was transferred, by female patient’s reporting area of residence^†^ at time of treatment — United States and Puerto Rico, 2017

Patient’s reporting area of residence	No. of ART clinics^§^	No. of ART procedures performed	No. of ART embryo-transfer procedures^¶^	No. of ART pregnancies	No. of ART live-birth deliveries	No. of ART singleton live-birth deliveries	No. of ART multiple live-birth deliveries	No. of ART live-born infants	ART procedures per 1 million women aged 15–44 yrs**
Alabama	5	969	736	400	326	265	61	391	1,020
Alaska	0	162	136	87	73	58	15	88	1,109
Arizona	15	2,956	2,428	1,334	1,095	876	219	1,321	2,197
Arkansas	1	607	468	234	199	146	53	253	1,051
California	68	24,179	19,425	10,779	8,852	7,789	1,063	9,926	2,983
Colorado	8	2,350	2,116	1,408	1,184	1,013	171	1,355	2,066
Connecticut	6	3,518	2,690	1,544	1,271	1,102	169	1,440	5,228
Delaware	2	744	535	304	219	214	5	224	4,126
District of Columbia	2	1,269	958	457	375	351	24	399	6,806
Florida	26	8,535	6,616	3,383	2,798	2,377	421	3,222	2,229
Georgia	8	4,264	3,673	2,014	1,657	1,440	217	1,875	1,986
Hawaii	5	1,010	802	430	341	258	83	426	3,798
Idaho	1	619	522	296	244	194	50	293	1,882
Illinois^††^	25	12,739	9,918	5,017	3,965	3,493	472	4,439	5,031
Indiana	9	2,227	1,786	841	682	562	120	804	1,719
Iowa	2	1,451	1,186	715	599	535	64	663	2,450
Kansas	4	1,001	830	469	383	337	46	429	1,792
Kentucky	4	1,431	1,168	534	435	364	71	505	1,687
Louisiana	5	1,517	1,135	600	502	434	68	571	1,621
Maine	1	513	441	229	191	167	24	216	2,216
Maryland	7	6,659	5,137	2,581	1,996	1,828	168	2,170	5,580
Massachusetts^††^	9	10,178	8,424	4,011	3,263	2,985	278	3,548	7,366
Michigan	11	3,939	3,184	1,730	1,426	1,130	296	1,728	2,102
Minnesota	5	3,066	2,694	1,499	1,239	1,061	178	1,418	2,874
Mississippi	2	579	485	262	220	196	24	245	979
Missouri	9	2,131	1,768	914	774	620	154	932	1,819
Montana	1	306	264	156	133	116	17	150	1,610
Nebraska	2	831	657	360	297	243	54	352	2,245
Nevada	5	1,181	996	571	447	388	59	509	2,005
New Hampshire	0	998	851	419	362	312	50	413	4,135
New Jersey^††^	19	10,562	8,317	4,799	3,940	3,599	341	4,285	6,158
New Mexico	2	367	342	185	159	132	27	187	928
New York	40	23,270	17,933	8,644	6,957	6,166	791	7,758	5,816
North Carolina	10	4,306	3,371	1,988	1,601	1,369	232	1,838	2,135
North Dakota	1	262	236	138	121	91	30	151	1,791
Ohio	11	4,687	3,727	2,002	1,637	1,443	194	1,837	2,127
Oklahoma	3	944	762	364	303	247	56	359	1,228
Oregon	3	1,273	1,144	752	641	525	116	761	1,571
Pennsylvania	15	7,082	5,586	2,883	2,317	2,084	233	2,554	2,971
Puerto Rico	3	222	194	96	67	49	18	85	338
Rhode Island^††^	1	950	806	328	273	250	23	296	4,544
South Carolina	4	1,713	1,315	695	566	483	83	645	1,774
South Dakota	1	282	242	134	111	92	19	130	1,775
Tennessee	10	1,789	1,434	797	662	572	90	756	1,363
Texas	41	14,594	11,849	6,581	5,374	4,577	797	6,185	2,480
Utah	3	2,184	1,923	1,116	925	764	161	1,087	3,235
Vermont	2	368	301	143	109	94	15	123	3,232
Virginia	10	6,149	4,924	2,649	2,137	1,916	221	2,360	3,658
Washington	12	3,909	3,053	1,830	1,517	1,362	155	1,672	2,669
West Virginia	3	341	270	123	96	77	19	115	1,058
Wisconsin	6	2,027	1,683	903	801	664	137	941	1,870
Wyoming	0	169	148	90	74	56	18	93	1,569
Non-U.S. resident	—	7,075	5,910	3,522	2,972	2,425	547	3,529	—^§§^
**Total**	**448**	**196,454**	**157,499**	**84,340**	**68,908**	**59,891**	**9,017**	**78,052**	**3,040**

**Abbreviation:** ART = assisted reproductive technology.

* Total number of cycles reported to CDC was 284,403. This report excludes 87,931 cycles in which egg or embryo banking was performed and 18 research cycles.

^†^ In cases of missing residency data (0.3%), the patient’s residence was assigned as the location where the ART procedure was performed.

^§^ The ART procedures and outcomes by patient’s reporting area of residence do not necessarily reflect the procedures and outcomes of the ART clinics within the reporting area because some patients seek treatment at a clinic in a location other than their area of residence.

^¶^ Embryo-transfer procedures include all procedures performed in which an attempt was made to transfer at least one embryo.

** On the basis of U.S. Census Bureau estimates (**Source:** US Census Bureau. Annual estimates of the resident population for selected age groups by sex for the United States, states, counties, and Puerto Rico Commonwealth and municipios: April 1, 2010 to July 1, 2017. Washington, DC: US Census Bureau, Population Division; 2017. https://data.census.gov/cedsci/table?q=United%20States&g=0100000US&tid=ACSST1Y2018.S0101&vintage=2018).

^††^ State with comprehensive insurance mandate requiring insurers to cover the costs associated with diagnosis and treatment of infertility inclusive of ART services for at least four oocyte retrievals.

^§§^ Non-U.S. residents were excluded from rate because the appropriate denominators were not available.

**FIGURE 2:**
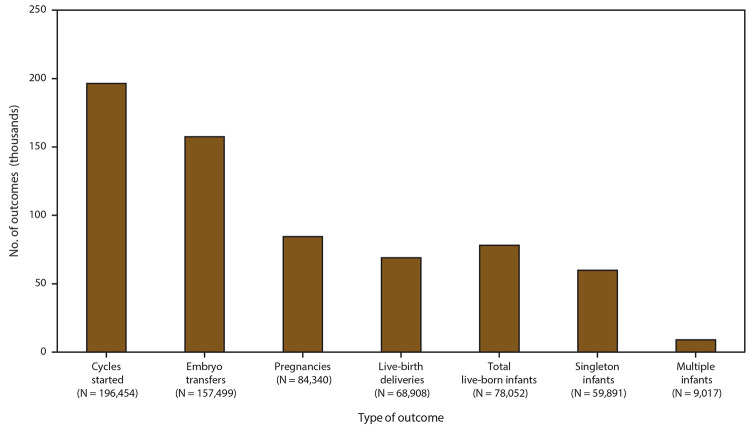
Number of outcomes of assisted reproductive technology procedures* with the intent to transfer at least one embryo, by type of outcome — United States and Puerto Rico, 2017 * A total of 284,403 assisted reproductive technology procedures were reported to CDC. Excluded are 87,931 cycles in which egg or embryo banking was performed and 18 research cycles in which a new treatment procedure was being evaluated.

In 2017, a total of 284,403 ART procedures were reported to CDC (*1*). Included in this report are data for 196,454 ART procedures performed (range: 162 in Alaska to 24,179 in California) in the United States (including Puerto Rico) with the intent to transfer at least one embryo (Table 1) (Figure 2). Excluded are 87,931 cycles in which egg or embryo banking was performed and 18 research cycles in which a new treatment procedure was being evaluated. Of 196,454 procedures performed in the 52 states or territories, 157,499 (80.2%) progressed to embryo transfer. Of 157,499 ART procedures that progressed to the embryo-transfer stage, 84,340 (53.5%) resulted in a pregnancy and 68,908 (43.8%) in a live-birth delivery (range: 67 in Puerto Rico to 8,852 in California). The 68,908 live-birth deliveries included 59,891 singleton deliveries (86.9%) and 9,017 multiple deliveries (13.1%) and resulted in 78,052 live-born infants (range: 85 in Puerto Rico to 9,926 in California).

Six states with the largest numbers of ART procedures (California, Illinois, Massachusetts, New Jersey, New York, and Texas) accounted for approximately half (48.6%; 95,522 of 196,454) of all ART procedures, 48.2% (75,866 of 157,499) of all embryo-transfer procedures, 46.3% (36,141 of 78,052) of all ART-conceived infants born, and 41.5% (3,742 of 9,017) of all ART-conceived multiple live-birth deliveries in the United States (Table 1). However, these six states accounted for only 36.2% of all U.S. births (*22*).

**FIGURE 3:**
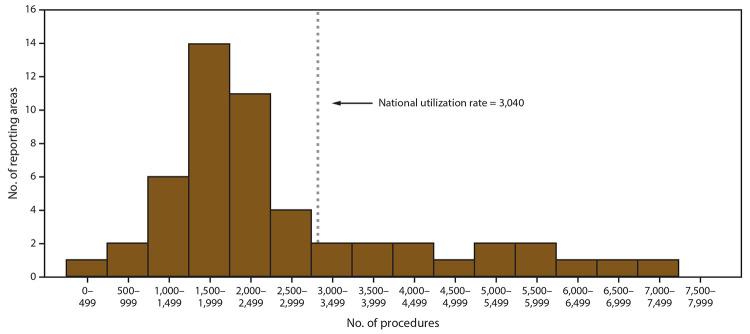
Number of reporting states and territories,* by number of assisted reproductive technology procedures performed^†^ among women of reproductive age (15–44 years)§ in which at least one embryo was transferred — United States and Puerto Rico, 2017^¶^ * Total number of reporting states and territories: 52. ^†^ Total number of procedures: 196,454. ^§^ Per 1 million women aged 15–44 years. ^¶^ National utilization rate = (Total number of procedures performed / Female population aged 15–44 years) X 1,000,000.

The number of ART procedures per 1 million women of reproductive age (15–44 years) ranged from 338 in Puerto Rico to 7,366 in Massachusetts, with an overall national rate of 3,040 (Table 1) (Figure 3). Fourteen states (Connecticut, Delaware, District of Columbia, Hawaii, Illinois, Maryland, Massachusetts, New Hampshire, New Jersey, New York, Rhode Island, Utah, Vermont, and Virginia) had ART use rates higher than the national rate. Of these, the District of Columbia (6,806), Massachusetts (7,366), and New Jersey (6,158) had rates exceeding twice the national rate, whereas Connecticut (5,228), Illinois (5,031), Maryland (5,580), and New York (5,816) had rates exceeding 1.5 times the national rate. The three areas with the lowest ART use rates were Puerto Rico (338), New Mexico (928), and Mississippi (979).

### Number of Embryos Transferred

The number of embryo-transfer procedures performed, the average number of embryos transferred per procedure, and the percentage of SET procedures among all embryo-transfer procedures performed are provided by state or territory and age group (Table 2). Overall, 65,387 embryo-transfer procedures were performed among women aged <35 years, 35,789 among women aged 35–37 years, and 56,323 among women aged >37 years. Nationally, on average, 1.3 embryos were transferred per procedure among women aged <35 years, 1.4 embryos among women aged 35–37 years, and 1.5 embryos among women aged >37 years. The national SET rate was 67.3% among women aged <35 years (range: 38.9% in South Dakota to 90.4% in Delaware), 65.0% among women aged 35–37 years (range: 23.6% in Puerto Rico to 89.4% in Delaware), and 60.0% among women aged >37 years (range: 28.6% in Puerto Rico to 83.1% in Delaware).

**TABLE 2:** Number of assisted reproductive technology embryo-transfer procedures with a single-embryo transfer,* by female patient’s age group and reporting area of residence^†^ at time of treatment — United States and Puerto Rico, 2017

Patient’s reporting area of residence	<35 yrs			35–37 yrs			>37 yrs		
	No. of embryo-transfer procedures	Average no. of embryos transferred	SET (%)	No. of embryo-transfer procedures	Average no. of embryos transferred	SET (%)	No. of embryo-transfer procedures	Average no. of embryos transferred	SET (%)
Alabama	388	1.5	55.4	149	1.5	54.4	199	1.7	48.2
Alaska	49	1.3	71.4	34	1.3	73.5	53	1.4	64.2
Arizona	1,124	1.6	47.8	574	1.6	51.4	730	1.7	50.7
Arkansas	296	1.5	50.3	90	1.6	43.3	82	1.5	52.4
California	6,030	1.3	68.4	4,637	1.3	68.5	8,758	1.5	63.3
Colorado	898	1.3	68.6	518	1.3	68.0	700	1.3	72.7
Connecticut	1,048	1.2	77.7	629	1.4	63.6	1,013	1.6	53.4
Delaware	250	1.1	90.4	113	1.1	89.4	172	1.2	83.1
District of Columbia	201	1.1	86.6	220	1.2	80.9	537	1.4	69.5
Florida	2,767	1.4	64.7	1,492	1.4	59.0	2,357	1.5	55.5
Georgia	1,528	1.3	71.1	843	1.4	66.3	1,302	1.4	66.5
Hawaii	253	1.6	40.7	197	1.6	39.6	352	1.9	36.9
Idaho	276	1.5	47.5	122	1.4	57.4	124	1.5	58.1
Illinois^§^	4,252	1.4	65.8	2,361	1.4	59.9	3,305	1.6	53.8
Indiana	1,001	1.4	57.8	360	1.5	55.3	425	1.5	54.6
Iowa	720	1.3	74.0	224	1.3	74.6	242	1.4	63.6
Kansas	473	1.3	73.6	173	1.3	70.5	184	1.3	70.1
Kentucky	707	1.5	56.4	214	1.5	58.9	247	1.6	47.4
Louisiana	596	1.4	61.9	275	1.4	56.4	264	1.5	61.4
Maine	225	1.3	72.9	97	1.4	66.0	119	1.3	72.3
Maryland	2,050	1.2	77.6	1,184	1.3	72.0	1,903	1.5	61.7
Massachusetts^§^	3,312	1.2	85.1	2,070	1.2	77.9	3,042	1.6	55.7
Michigan	1,574	1.6	44.7	702	1.6	45.6	908	1.7	46.1
Minnesota	1,334	1.4	63.0	602	1.4	58.5	758	1.4	61.1
Mississippi	269	1.3	75.8	98	1.5	53.1	118	1.5	61.0
Missouri	1,004	1.5	50.2	398	1.5	52.3	366	1.7	48.6
Montana	120	1.3	71.7	62	1.3	71.0	82	1.5	61.0
Nebraska	395	1.4	57.2	125	1.5	55.2	137	1.5	58.4
Nevada	463	1.4	58.7	198	1.3	70.7	335	1.4	66.3
New Hampshire	399	1.2	76.2	230	1.3	71.7	222	1.6	54.1
New Jersey^§^	3,277	1.2	78.8	1,950	1.3	73.7	3,090	1.4	69.8
New Mexico	155	1.4	65.8	83	1.3	72.3	104	1.4	66.3
New York	6,227	1.3	68.4	3,780	1.4	64.4	7,926	1.6	57.4
North Carolina	1,557	1.3	68.3	813	1.4	62.2	1,001	1.5	55.5
North Dakota	150	1.5	49.3	46	1.4	60.9	40	1.5	57.5
Ohio	1,960	1.3	67.3	834	1.4	61.5	933	1.6	52.0
Oklahoma	451	1.5	47.0	138	1.7	37.0	173	1.8	32.9
Oregon	427	1.5	53.9	294	1.4	60.9	423	1.4	65.7
Pennsylvania	2,572	1.3	73.7	1,336	1.3	68.1	1,678	1.4	64.8
Puerto Rico	55	1.7	41.8	55	1.8	23.6	84	2.0	28.6
Rhode Island^§^	326	1.2	81.6	193	1.3	72.0	287	1.8	44.3
South Carolina	661	1.3	66.1	301	1.4	59.5	353	1.5	55.2
South Dakota	167	1.6	38.9	48	1.6	37.5	27	1.6	48.1
Tennessee	689	1.4	65.5	342	1.5	58.5	403	1.5	58.6
Texas	5,537	1.4	64.2	2,741	1.4	63.1	3,571	1.5	60.8
Utah	1,158	1.4	59.2	354	1.5	55.1	411	1.5	55.7
Vermont	132	1.3	68.9	81	1.4	59.3	88	1.6	52.3
Virginia	1,863	1.3	74.7	1,161	1.3	72.1	1,900	1.4	66.8
Washington	1,218	1.3	72.7	736	1.3	75.0	1,099	1.3	73.4
West Virginia	161	1.5	55.3	55	1.4	61.8	54	1.4	63.0
Wisconsin	884	1.4	60.5	385	1.4	57.9	414	1.6	48.6
Wyoming	77	1.4	55.8	34	1.6	44.1	37	1.4	59.5
Non-U.S. resident	1,681	1.4	61.7	1,038	1.4	62.4	3,191	1.4	62.9
**Total**	**65,387**	**1.3**	**67.3**	**35,789**	**1.4**	**65.0**	**56,323**	**1.5**	**60.0**

**Abbreviation:** SET = single-embryo transfer.

* Includes all procedures in which at least one embryo was transferred.

^†^ In cases of missing residency data (0.3%), the patient’s residence was assigned as the location where the assisted reproductive technology procedure was performed.

^§^ State with comprehensive insurance mandate requiring insurers to cover the costs associated with diagnosis and treatment of infertility inclusive of ART services for at least four oocyte retrievals.

### Singleton and Multiple Births 

In 2017, among 3,879,810 infants born in the United States and Puerto Rico, 74,006 (1.9%) were conceived with ART procedures performed in 2016 and 2017 (Table 3). California, Texas, and New York had the highest total numbers of all infants born (471,658; 382,050; and 229,737, respectively) and ART-conceived infants born (9,844; 6,164; and 7,495, respectively). The percentage of ART-conceived infants among all infants born was highest in Massachusetts (5.0%), followed by the District of Columbia and Connecticut (4.4% and 4.3%, respectively).

**TABLE 3:** Number, proportion, and percentage of infants born with use of assisted reproductive technology, by female patient’s reporting area of residence* at time of treatment — United States and Puerto Rico, 2017^†^

Patient’s reporting area of residence	Total no. of infants born^§,¶^	No. of ART infants born	Proportion of ART infants among all infants (%)	Singleton infants		
				ART infants	All infants	Proportion of ART singleton infants among all singleton infants (%)
				No. (%)	No. (%)	
Alabama	58,941	388	0.7	253 (65.2)	56,770 (96.3)	0.4
Alaska	10,445	81	0.8	61 (75.3)	10,142 (97.1)	0.6
Arizona	81,872	1,424	1.7	871 (61.2)	79,200 (96.7)	1.1
Arkansas	37,520	245	0.7	150 (61.2)	36,306 (96.8)	0.4
California	471,658	9,844	2.1	7,459 (75.8)	456,782 (96.8)	1.6
Colorado	64,382	1,298	2.0	937 (72.2)	62,346 (96.8)	1.5
Connecticut	35,221	1,525	4.3	1,097 (71.9)	33,769 (95.9)	3.2
Delaware	10,855	248	2.3	224 (90.3)	10,473 (96.5)	2.1
District of Columbia	9,560	416	4.4	374 (89.9)	9,200 (96.2)	4.1
Florida	223,630	3,296	1.5	2,248 (68.2)	216,180 (96.7)	1.0
Georgia	129,243	1,771	1.4	1,339 (75.6)	124,669 (96.5)	1.1
Hawaii	17,517	461	2.6	268 (58.1)	16,908 (96.5)	1.6
Idaho	22,181	265	1.2	164 (61.9)	21,478 (96.8)	0.8
Illinois**	149,390	4,463	3.0	3,326 (74.5)	143,869 (96.3)	2.3
Indiana	82,170	796	1.0	545 (68.5)	79,380 (96.6)	0.7
Iowa	38,430	642	1.7	505 (78.7)	37,157 (96.7)	1.4
Kansas	36,519	453	1.2	325 (71.7)	35,285 (96.6)	0.9
Kentucky	54,752	527	1.0	357 (67.7)	52,879 (96.6)	0.7
Louisiana	61,018	624	1.0	403 (64.6)	58,853 (96.5)	0.7
Maine	12,298	215	1.7	160 (74.4)	11,911 (96.9)	1.3
Maryland	71,641	2,196	3.1	1,802 (82.1)	69,103 (96.5)	2.6
Massachusetts**	70,702	3,557	5.0	2,974 (83.6)	68,132 (96.4)	4.4
Michigan	111,426	1,689	1.5	1,032 (61.1)	107,123 (96.1)	1.0
Minnesota	68,595	1,387	2.0	990 (71.4)	66,177 (96.5)	1.5
Mississippi	37,357	237	0.6	168 (70.9)	36,089 (96.6)	0.5
Missouri	73,034	1,021	1.4	677 (66.3)	70,323 (96.3)	1.0
Montana	11,799	152	1.3	96 (63.2)	11,414 (96.7)	0.8
Nebraska	25,821	369	1.4	245 (66.4)	24,816 (96.1)	1.0
Nevada	35,756	575	1.6	401 (69.7)	34,623 (96.8)	1.2
New Hampshire	12,116	306	2.5	248 (81.0)	11,663 (96.3)	2.1
New Jersey**	101,250	4,176	4.1	3,365 (80.6)	97,500 (96.3)	3.5
New Mexico	23,767	155	0.7	100 (64.5)	23,142 (97.4)	0.4
New York	229,737	7,495	3.3	5,763 (76.9)	221,332 (96.3)	2.6
North Carolina	120,125	1,890	1.6	1,281 (67.8)	115,762 (96.4)	1.1
North Dakota	10,737	154	1.4	84 (54.5)	10,343 (96.3)	0.8
Ohio	136,832	1,827	1.3	1,276 (69.8)	131,943 (96.4)	1.0
Oklahoma	50,214	389	0.8	251 (64.5)	48,651 (96.9)	0.5
Oregon	43,631	757	1.7	473 (62.5)	42,073 (96.4)	1.1
Pennsylvania	137,745	2,593	1.9	1,984 (76.5)	132,873 (96.5)	1.5
Puerto Rico	24,310	88	0.4	48 (54.5)	23,834 (98.0)	0.2
Rhode Island**	10,638	295	2.8	215 (72.9)	10,267 (96.5)	2.1
South Carolina	57,029	660	1.2	466 (70.6)	55,029 (96.5)	0.8
South Dakota	12,134	115	0.9	85 (73.9)	11,693 (96.4)	0.7
Tennessee	81,016	753	0.9	564 (74.9)	78,394 (96.8)	0.7
Texas	382,050	6,164	1.6	4,254 (69.0)	369,730 (96.8)	1.2
Utah	48,585	1,005	2.1	681 (67.8)	46,831 (96.4)	1.5
Vermont	5,655	132	2.3	94 (71.2)	5,474 (96.8)	1.7
Virginia	100,391	2,130	2.1	1,710 (80.3)	96,871 (96.5)	1.8
Washington	87,562	1,594	1.8	1,278 (80.2)	84,864 (96.9)	1.5
West Virginia	18,675	140	0.7	93 (66.4)	18,076 (96.8)	0.5
Wisconsin	64,975	946	1.5	620 (65.5)	62,671 (96.5)	1.0
Wyoming	6,903	77	1.1	52 (67.5)	6,734 (97.6)	0.8
**Total**	**3,879,810**	**74,006**	**1.9**	**54,436 (73.6)**	**3,747,107 (96.6)**	**1.5**

**Abbreviation:** ART = assisted reproductive technology.

* In cases of missing residency data (0.3%), the patient’s residence was assigned as the location where the ART procedure was performed.

^†^ Includes infants conceived from ART procedures performed in 2016 and born in 2017 and infants conceived from ART procedures performed in 2017 and born in 2017. Total ART births exclude births to non-U.S. residents.

^§^ U.S. births include births to non-U.S. residents (**Source:** Martin JA, Hamilton BE, Osterman MJ, Driscoll AK, Drake P. Births: final data for 2017. Natl Vital Stat Rep 2018;67:1–50).

^¶^ U.S. births include births to non-U.S. residents (**Source:** CDC Wonder [Internet]. Natality public use data 2007–2017. Atlanta, GA: US Department of Health and Human Services, CDC; 2018).

** State with comprehensive insurance mandate requiring insurers to cover the costs associated with diagnosis and treatment of infertility inclusive of ART services for at least four oocyte retrievals.

Nationally, 26.4% of ART-conceived infants were born in multiple-birth deliveries (range: 9.7% in Delaware to 45.5% in North Dakota and Puerto Rico), compared with 3.4% of all infants (range: 2.0% in Puerto Rico to 4.1% in Connecticut) (Table 4). ART-conceived twins accounted for approximately 96.5% (18,890 of 19,570) of all ART-conceived infants born in multiple-birth deliveries. ART-conceived multiple-birth infants contributed to 14.7% of all multiple births (range: 5.4% in Mississippi to 31.7% in Hawaii). Approximately 25.5% of all ART-conceived infants were twins, compared with 3.3% of all infants. ART-conceived twins contributed to 14.7% of all twins. Of ART-conceived infants, 0.9% were triplets and higher-order multiples, compared with 0.1% among all infants. ART-conceived triplets and higher-order infants contributed to 17.3% of all triplets and higher-order infants.

**TABLE 4:** Number, percentage, and proportion of multiple-birth infants, twins, and triplets and higher-order infants born with use of assisted reproductive technology procedures, by female patient’s reporting area of residence* at time of treatment — United States and Puerto Rico, 2017^†^

Patient’s reporting area of residence	Multiple-birth infants			Twin infants			Triplets and higher-order infants		
	ART infants^§^	All infants^¶^	Proportion of ART multiple births among all multiple births (%)	ART infants^§^	All infants^¶^	Proportion of ART twin infants among all twin infants (%)	ART infants^§^	All infants^¶^	Proportion of ART triplets and higher-order infants among all triplets and higher-order infants (%)
	No. (%)	No. (%)		No. (%)	No. (%)		No. (%)	No. (%)	
Alabama	135 (34.8)	2,171 (3.7)	6.2	129 (33.2)	2,068 (3.5)	6.2	6 (1.5)	103 (0.2)	5.8
Alaska	20 (24.7)	—** (—)	—**	20 (24.7)	297 (2.8)	6.7	0 (0.0)	—** (—)	—^††^
Arizona	553 (38.8)	2,672 (3.3)	20.7	525 (36.9)	2,581 (3.2)	20.3	28 (2.0)	91 (0.1)	30.8
Arkansas	95 (38.8)	1,214 (3.2)	7.8	—** (—)	1,193 (3.2)	—**	—** (—)	21 (0.1)	—**
California	2,385 (24.2)	14,876 (3.2)	16.0	2,320 (23.6)	14,495 (3.1)	16.0	65 (0.7)	381 (0.1)	17.1
Colorado	361 (27.8)	2,036 (3.2)	17.7	346 (26.7)	1,985 (3.1)	17.4	15 (1.2)	51 (0.1)	29.4
Connecticut	428 (28.1)	1,452 (4.1)	29.5	419 (27.5)	1,426 (4.0)	29.4	9 (0.6)	26 (0.1)	34.6
Delaware	24 (9.7)	—** (—)	—**	24 (9.7)	379 (3.5)	6.3	0 (0.0)	—** (—)	—^††^
District of Columbia	42 (10.1)	—** (—)	—**	42 (10.1)	358 (3.7)	11.7	0 (0.0)	—** (—)	—^††^
Florida	1,048 (31.8)	7,450 (3.3)	14.1	1,031 (31.3)	7,288 (3.3)	14.1	17 (0.5)	162 (0.1)	10.5
Georgia	432 (24.4)	4,574 (3.5)	9.4	413 (23.3)	4,456 (3.4)	9.3	19 (1.1)	118 (0.1)	16.1
Hawaii	193 (41.9)	609 (3.5)	31.7	184 (39.9)	597 (3.4)	30.8	9 (2.0)	12 (0.1)	—^††^
Idaho	101 (38.1)	703 (3.2)	14.4	—** (—)	691 (3.1)	—**	—** (—)	12 (0.1)	—**^,††^
Illinois	1,137 (25.5)	5,521 (3.7)	20.6	1,105 (24.8)	5,337 (3.6)	20.7	32 (0.7)	184 (0.1)	17.4
Indiana	251 (31.5)	2,790 (3.4)	9.0	236 (29.6)	2,660 (3.2)	8.9	15 (1.9)	130 (0.2)	11.5
Iowa	137 (21.3)	1,273 (3.3)	10.8	137 (21.3)	1,234 (3.2)	11.1	0 (0.0)	39 (0.1)	0.0
Kansas	128 (28.3)	1,234 (3.4)	10.4	128 (28.3)	1,216 (3.3)	10.5	0 (0.0)	18 (0.0)	—^††^
Kentucky	170 (32.3)	1,873 (3.4)	9.1	164 (31.1)	1,834 (3.3)	8.9	6 (1.1)	39 (0.1)	15.4
Louisiana	221 (35.4)	2,165 (3.5)	10.2	209 (33.5)	2,099 (3.4)	10.0	12 (1.9)	66 (0.1)	18.2
Maine	55 (25.6)	—** (—)	—**	55 (25.6)	378 (3.1)	14.6	0 (0.0)	—** (—)	—^††^
Maryland	394 (17.9)	2,538 (3.5)	15.5	367 (16.7)	2,457 (3.4)	14.9	27 (1.2)	81 (0.1)	33.3
Massachusetts	583 (16.4)	2,570 (3.6)	22.7	568 (16.0)	2,523 (3.6)	22.5	15 (0.4)	47 (0.1)	31.9
Michigan	657 (38.9)	4,303 (3.9)	15.3	642 (38.0)	4,162 (3.7)	15.4	15 (0.9)	141 (0.1)	10.6
Minnesota	397 (28.6)	2,418 (3.5)	16.4	384 (27.7)	2,347 (3.4)	16.4	13 (0.9)	71 (0.1)	18.3
Mississippi	69 (29.1)	1,268 (3.4)	5.4	—** (—)	1,231 (3.3)	—**	—** (—)	37 (0.1)	—**
Missouri	344 (33.7)	2,711 (3.7)	12.7	326 (31.9)	2,615 (3.6)	12.5	18 (1.8)	96 (0.1)	18.8
Montana	56 (36.8)	—** (—)	—**	56 (36.8)	382 (3.2)	14.7	0 (0.0)	—** (—)	—^††^
Nebraska	124 (33.6)	1,005 (3.9)	12.3	118 (32.0)	953 (3.7)	12.4	6 (1.6)	52 (0.2)	11.5
Nevada	174 (30.3)	1,133 (3.2)	15.4	165 (28.7)	1,100 (3.1)	15.0	9 (1.6)	33 (0.1)	27.3
New Hampshire	58 (19.0)	453 (3.7)	12.8	52 (17.0)	437 (3.6)	11.9	6 (2.0)	16 (0.1)	—^††^
New Jersey	811 (19.4)	3,750 (3.7)	21.6	780 (18.7)	3,642 (3.6)	21.4	31 (0.7)	108 (0.1)	28.7
New Mexico	55 (35.5)	625 (2.6)	8.8	46 (29.7)	600 (2.5)	7.7	9 (5.8)	25 (0.1)	36.0
New York	1,732 (23.1)	8,405 (3.7)	20.6	1,678 (22.4)	8,111 (3.5)	20.7	54 (0.7)	294 (0.1)	18.4
North Carolina	609 (32.2)	4,363 (3.6)	14.0	586 (31.0)	4,241 (3.5)	13.8	23 (1.2)	122 (0.1)	18.9
North Dakota	70 (45.5)	394 (3.7)	17.8	70 (45.5)	377 (3.5)	18.6	0 (0.0)	17 (0.2)	—^††^
Ohio	551 (30.2)	4,889 (3.6)	11.3	530 (29.0)	4,741 (3.5)	11.2	21 (1.1)	148 (0.1)	14.2
Oklahoma	138 (35.5)	1,563 (3.1)	8.8	—** (—)	1,523 (3.0)	—**	—** (—)	40 (0.1)	—**
Oregon	284 (37.5)	1,558 (3.6)	18.2	272 (35.9)	1,515 (3.5)	18.0	12 (1.6)	43 (0.1)	27.9
Pennsylvania	609 (23.5)	4,872 (3.5)	12.5	591 (22.8)	4,738 (3.4)	12.5	18 (0.7)	134 (0.1)	13.4
Puerto Rico	40 (45.5)	476 (2.0)	8.4	34 (38.6)	464 (1.9)	7.3	6 (6.8)	12 (0.0)	—^††^
Rhode Island	80 (27.1)	—** (—)	—**	80 (27.1)	363 (3.4)	22.0	0 (0.0)	—** (—)	—^††^
South Carolina	194 (29.4)	2,000 (3.5)	9.7	—** (—)	1,950 (3.4)	—**	—** (—)	50 (0.1)	—**
South Dakota	30 (26.1)	441 (3.6)	6.8	30 (26.1)	427 (3.5)	7.0	0 (0.0)	14 (0.1)	—^††^
Tennessee	189 (25.1)	2,622 (3.2)	7.2	—** (—)	2,538 (3.1)	—**	—** (—)	84 (0.1)	—**
Texas	1,910 (31.0)	12,320 (3.2)	15.5	1,822 (29.6)	11,926 (3.1)	15.3	88 (1.4)	394 (0.1)	22.3
Utah	324 (32.2)	1,754 (3.6)	18.5	309 (30.7)	1,690 (3.5)	18.3	15 (1.5)	64 (0.1)	23.4
Vermont	38 (28.8)	—** (—)	—**	38 (28.8)	176 (3.1)	21.6	0 (0.0)	—** (—)	—^††^
Virginia	420 (19.7)	3,520 (3.5)	11.9	408 (19.2)	3,410 (3.4)	12.0	12 (0.6)	110 (0.1)	10.9
Washington	316 (19.8)	2,698 (3.1)	11.7	303 (19.0)	2,607 (3.0)	11.6	13 (0.8)	91 (0.1)	14.3
West Virginia	47 (33.6)	599 (3.2)	7.8	—** (—)	576 (3.1)	—**	—** (—)	23 (0.1)	—**
Wisconsin	326 (34.5)	2,304 (3.5)	14.1	314 (33.2)	2,224 (3.4)	14.1	12 (1.3)	80 (0.1)	15.0
Wyoming	25 (32.5)	169 (2.4)	14.8	—** (—)	156 (2.3)	—**	—** (—)	13 (0.2)	—**^,††^
**Total**	**19,570 (26.4)**	**132,703 (3.4)**	**14.7**	**18,890 (25.5)**	**128,774 (3.3)**	**14.7**	**680 (0.9)**	**3,929 (0.1)**	**17.3**

**Abbreviation:** ART = assisted reproductive technology.

* In cases of missing residency data (0.3%), the patient’s residence was assigned as the location where the ART procedure was performed.

^†^ ART totals include infants conceived from ART procedures performed in 2016 and born in 2017 and infants conceived from ART procedures performed in 2017 and born in 2017. Total ART births exclude births to non-U.S. residents.

^§^ Includes only the number of infants live born in a multiple-birth delivery. For example, if three infants were born in a live-birth delivery and one of the three infants was stillborn, the total number of live-born infants would be two. However, the two infants still would be counted as triplets.

^¶^ U.S. births include births to non-U.S. residents (**Source:** CDC Wonder [Internet]. Natality public use data 2007–2017. Atlanta, GA: US Department of Health and Human Services, CDC; 2018).

** To protect confidentiality, cells with values of 1–4 for ART infants and cells with values of 0–9 for all infants are suppressed. Also suppressed are data that can be used to derive suppressed cell values. These values are included in the totals.

^††^ Estimates on the basis of N <20 in the denominator have been suppressed because such rates are considered unstable.

### Adverse Perinatal Outcomes

Nationally, ART-conceived infants contributed to 4.5% of all infants with low birthweight, 4.5% of all infants with moderately low birthweight, and 4.6% of all infants with very low birthweight (Table 5). Among all ART-conceived infants (including multiples), 20.2% had low birthweight, compared with 8.3% among all infants (including multiples). Approximately 3.5% of all ART-conceived infants (including multiples) had very low birthweight, compared with 1.4% among all infants (including multiples).

**TABLE 5:** Number, percentage, and proportion of infants born with use of assisted reproductive technology,* by low birthweight category and female patient’s reporting area of residence^†^ at time of treatment — United States and Puerto Rico, 2017

Patient’s reporting area of residence	<1,500 g (VLBW)			1,500–2,499 g (MLBW)			<2,500 g (LBW)		
	ART infants	All infants^§^	Proportion of ART VLBW infants among all VLBW infants (%)	ART infants	All infants^§^	Proportion of ART MLBW infants among all MLBW infants (%)	ART infants	All infants^§^	Proportion of ART LBW infants among all LBW infants (%)
	No. (%)	No. (%)		No. (%)	No. (%)		No. (%)	No. (%)	
Alabama	16 (4.2)	1,119 (1.9)	1.4	92 (23.9)	4,919 (8.3)	1.9	108 (28.1)	6,038 (10.2)	1.8
Alaska	—^¶^ (—)	104 (1.0)	—	— (—)	543 (5.2)	—	19 (23.5)	647 (6.2)	2.9
Arizona	41 (3.0)	952 (1.2)	4.3	298 (21.9)	5,167 (6.3)	5.8	339 (24.9)	6,119 (7.5)	5.5
Arkansas	9 (3.7)	598 (1.6)	1.5	64 (26.4)	2,879 (7.7)	2.2	73 (30.2)	3,477 (9.3)	2.1
California	273 (2.9)	5,191 (1.1)	5.3	1,472 (15.4)	27,260 (5.8)	5.4	1,745 (18.3)	32,451 (6.9)	5.4
Colorado	49 (3.9)	779 (1.2)	6.3	270 (21.5)	5,069 (7.9)	5.3	319 (25.4)	5,848 (9.1)	5.5
Connecticut	75 (5.0)	513 (1.5)	14.6	245 (16.3)	2,332 (6.6)	10.5	320 (21.3)	2,845 (8.1)	11.2
Delaware	— (—)	169 (1.6)	—	— (—)	812 (7.5)	—	33 (13.7)	981 (9.0)	3.4
District of Columbia	— (—)	206 (2.2)	—	— (—)	795 (8.3)	—	49 (12.0)	1,001 (10.5)	4.9
Florida	113 (3.5)	3,433 (1.5)	3.3	613 (19.2)	16,220 (7.3)	3.8	726 (22.7)	19,653 (8.8)	3.7
Georgia	66 (3.8)	2,319 (1.8)	2.8	292 (16.7)	10,453 (8.1)	2.8	358 (20.5)	12,772 (9.9)	2.8
Hawaii	16 (3.6)	224 (1.3)	7.1	108 (24.6)	1,267 (7.2)	8.5	124 (28.2)	1,491 (8.5)	8.3
Idaho	9 (3.5)	231 (1.0)	3.9	56 (21.5)	1,314 (5.9)	4.3	65 (25.0)	1,545 (7.0)	4.2
Illinois	156 (3.6)	2,131 (1.4)	7.3	709 (16.1)	10,520 (7.0)	6.7	865 (19.7)	12,651 (8.5)	6.8
Indiana	26 (3.3)	1,152 (1.4)	2.3	148 (19.0)	5,642 (6.9)	2.6	174 (22.3)	6,794 (8.3)	2.6
Iowa	18 (2.8)	422 (1.1)	4.3	85 (13.4)	2,104 (5.5)	4.0	103 (16.2)	2,526 (6.6)	4.1
Kansas	16 (3.7)	476 (1.3)	3.4	68 (15.8)	2,209 (6.0)	3.1	84 (19.5)	2,685 (7.4)	3.1
Kentucky	17 (3.3)	827 (1.5)	2.1	93 (18.0)	4,004 (7.3)	2.3	110 (21.3)	4,831 (8.8)	2.3
Louisiana	29 (4.8)	1,138 (1.9)	2.5	147 (24.1)	5,381 (8.8)	2.7	176 (28.9)	6,519 (10.7)	2.7
Maine	— (—)	147 (1.2)	—	— (—)	729 (5.9)	—	41 (19.2)	876 (7.1)	4.7
Maryland	90 (4.1)	1,261 (1.8)	7.1	301 (13.8)	5,114 (7.1)	5.9	391 (17.9)	6,375 (8.9)	6.1
Massachusetts	65 (1.9)	772 (1.1)	8.4	430 (12.5)	4,488 (6.3)	9.6	495 (14.4)	5,260 (7.4)	9.4
Michigan	62 (3.7)	1,637 (1.5)	3.8	340 (20.6)	8,156 (7.3)	4.2	402 (24.3)	9,793 (8.8)	4.1
Minnesota	51 (3.7)	810 (1.2)	6.3	216 (15.7)	3,816 (5.6)	5.7	267 (19.5)	4,626 (6.7)	5.8
Mississippi	5 (2.1)	780 (2.1)	0.6	39 (16.7)	3,553 (9.5)	1.1	44 (18.9)	4,333 (11.6)	1.0
Missouri	44 (4.6)	1,087 (1.5)	4.0	190 (19.7)	5,249 (7.2)	3.6	234 (24.2)	6,336 (8.7)	3.7
Montana	— (—)	122 (1.0)	—	— (—)	820 (6.9)	—	36 (24.0)	942 (8.0)	3.8
Nebraska	6 (1.7)	297 (1.2)	2.0	78 (21.7)	1,633 (6.3)	4.8	84 (23.3)	1,930 (7.5)	4.4
Nevada	20 (3.6)	530 (1.5)	3.8	98 (17.6)	2,735 (7.6)	3.6	118 (21.1)	3,265 (9.1)	3.6
New Hampshire	5 (1.7)	125 (1.0)	4.0	40 (13.2)	714 (5.9)	5.6	45 (14.9)	839 (6.9)	5.4
New Jersey	128 (3.2)	1,366 (1.3)	9.4	592 (14.6)	6,674 (6.6)	8.9	720 (17.8)	8,040 (7.9)	9.0
New Mexico	18 (11.8)	311 (1.3)	5.8	41 (27.0)	1,939 (8.2)	2.1	59 (38.8)	2,250 (9.5)	2.6
New York	209 (3.0)	3,200 (1.4)	6.5	1,089 (15.5)	15,343 (6.7)	7.1	1,298 (18.4)	18,543 (8.1)	7.0
North Carolina	77 (4.4)	2,025 (1.7)	3.8	311 (17.7)	9,243 (7.7)	3.4	388 (22.1)	11,268 (9.4)	3.4
North Dakota	13 (8.4)	130 (1.2)	10.0	22 (14.3)	590 (5.5)	3.7	35 (22.7)	720 (6.7)	4.9
Ohio	66 (3.7)	2,107 (1.5)	3.1	315 (17.5)	9,747 (7.1)	3.2	381 (21.2)	11,854 (8.7)	3.2
Oklahoma	19 (5.0)	748 (1.5)	2.5	66 (17.4)	3,337 (6.6)	2.0	85 (22.4)	4,085 (8.1)	2.1
Oregon	20 (2.8)	449 (1.0)	4.5	150 (20.8)	2,523 (5.8)	5.9	170 (23.5)	2,972 (6.8)	5.7
Pennsylvania	73 (2.9)	2,092 (1.5)	3.5	399 (15.9)	9,488 (6.9)	4.2	472 (18.8)	11,580 (8.4)	4.1
Puerto Rico	6 (7.2)	347 (1.4)	1.7	25 (30.1)	2,209 (9.1)	1.1	31 (37.3)	2,556 (10.5)	1.2
Rhode Island	13 (4.5)	158 (1.5)	8.2	41 (14.3)	637 (6.0)	6.4	54 (18.8)	795 (7.5)	6.8
South Carolina	18 (2.9)	1,004 (1.8)	1.8	105 (17.1)	4,502 (7.9)	2.3	123 (20.0)	5,506 (9.7)	2.2
South Dakota	— (—)	142 (1.2)	—	— (—)	693 (5.7)	—	18 (16.2)	835 (6.9)	2.2
Tennessee	12 (1.6)	1,224 (1.5)	1.0	124 (16.9)	6,185 (7.6)	2.0	136 (18.6)	7,409 (9.1)	1.8
Texas	295 (4.9)	5,437 (1.4)	5.4	1,150 (19.1)	26,725 (7.0)	4.3	1,445 (24.0)	32,162 (8.4)	4.5
Utah	61 (6.2)	589 (1.2)	10.4	203 (20.6)	2,918 (6.0)	7.0	264 (26.8)	3,507 (7.2)	7.5
Vermont	6 (4.6)	62 (1.1)	9.7	13 (9.9)	318 (5.6)	4.1	19 (14.5)	380 (6.7)	5.0
Virginia	74 (3.6)	1,526 (1.5)	4.8	297 (14.3)	6,867 (6.8)	4.3	371 (17.8)	8,393 (8.4)	4.4
Washington	31 (2.0)	860 (1.0)	3.6	224 (14.2)	4,916 (5.6)	4.6	255 (16.2)	5,776 (6.6)	4.4
West Virginia	— (—)	295 (1.6)	—	— (—)	1,486 (8.0)	—	31 (23.0)	1,781 (9.5)	1.7
Wisconsin	36 (3.9)	795 (1.2)	4.5	163 (17.5)	4,173 (6.4)	3.9	199 (21.4)	4,968 (7.6)	4.0
Wyoming	7 (9.5)	63 (0.9)	11.1	9 (12.2)	537 (7.8)	1.7	16 (21.6)	600 (8.7)	2.7
**Total**	**2,486 (3.5)**	**54,482 (1.4)**	**4.6**	**12,031 (16.8)**	**266,947 (6.9)**	**4.5**	**14,517 (20.2)**	**321,429 (8.3)**	**4.5**

**Abbreviations:** ART = assisted reproductive technology; LBW = low birthweight; MLBW = moderately low birthweight; VLBW = very low birthweight.

* ART totals include infants conceived from ART procedures performed in 2016 and born in 2017 and infants conceived from ART procedures performed in 2017 and born in 2017. Total ART infants exclude births to non-U.S. residents and include only infants with birthweight data available.

^†^ In cases of missing residency data (0.3%), the patient’s residence was assigned as the location where the ART procedure was performed.

^§^ U.S. births include births to non-U.S. residents (**Source:** CDC Wonder [Internet]. Natality public use data 2007–2017. Atlanta, GA: US Department of Health and Human Services, CDC; 2018).

^¶^ To protect confidentiality, cells with values of 1–4 for ART infants and cells with values of 0–9 for all infants are suppressed. Also suppressed are data that can be used to derive suppressed cell values. These values are included in the totals.

Nationally, ART contributed to approximately 5.3% of all infants born very preterm, 6.0% early preterm, 5.1% late preterm, and 5.3% preterm (Table 6). In Connecticut, Massachusetts, and New Jersey, the contribution of ART to preterm infants exceeded 10% in all categories of preterm birth. Among all ART-conceived infants (including multiples), rates for preterm birth were 4.5% very preterm, 8.7% early preterm, 19.2% late preterm, and 27.8% preterm. Corresponding rates of preterm birth among all infants (including multiples) born were 1.6% very preterm, 2.8% early preterm, 7.2% late preterm, and 9.9% preterm. Late preterm births accounted for the majority of preterm births both among ART-conceived infants and all infants (69.0% and 72.0%, respectively).

**TABLE 6:** Number, percentage, and proportion of infants born with use of assisted reproductive technology,* by preterm gestational age category and female patient’s reporting area of residence^†^ at time of treatment — United States and Puerto Rico, 2017

Patient’s reporting area of residence	VPTB (<32 wks)			Early PTB (<34 wks)			Late PTB (34–36 wks)			PTB (<37 wks)		
	ART infants	All infants^§^	Proportion of ART VPTB infants among all VPTB infants (%)	ART infants	All infants^§^	Proportion of ART early PTB infants among all early PTB infants (%)	ART infants	All infants^§^	Proportion of ART late PTB infants among all late PTB infants (%)	ART infants	All infants^§^	Proportion of ART PTB infants among all PTB infants (%)
	No. (%)	No. (%)		No. (%)	No. (%)		No. (%)	No. (%)		No. (%)	No. (%)	
Alabama	25 (6.5)	1,167 (2.0)	2.1	40 (10.3)	2,001 (3.4)	2.0	111 (28.7)	5,089 (8.6)	2.2	151 (39.0)	7,090 (12.0)	2.1
Alaska	6 (7.4)	134 (1.3)	4.5	8 (9.9)	259 (2.5)	3.1	13 (16.0)	678 (6.5)	1.9	21 (25.9)	937 (9.0)	2.2
Arizona	60 (4.2)	1,090 (1.3)	5.5	124 (8.7)	1,956 (2.4)	6.3	372 (26.2)	5,622 (6.9)	6.6	496 (34.9)	7,578 (9.3)	6.5
Arkansas	17 (6.9)	673 (1.8)	2.5	36 (14.7)	1,165 (3.1)	3.1	69 (28.2)	3,103 (8.3)	2.2	105 (42.9)	4,268 (11.4)	2.5
California	380 (3.9)	5,999 (1.3)	6.3	744 (7.6)	10,683 (2.3)	7.0	1,621 (16.6)	30,224 (6.4)	5.4	2,365 (24.2)	40,907 (8.7)	5.8
Colorado	64 (5.0)	831 (1.3)	7.7	136 (10.6)	1,536 (2.4)	8.9	278 (21.6)	4,102 (6.4)	6.8	414 (32.2)	5,638 (8.8)	7.3
Connecticut	93 (6.1)	580 (1.6)	16.0	152 (10.0)	982 (2.8)	15.5	251 (16.5)	2,356 (6.7)	10.7	403 (26.5)	3,338 (9.5)	12.1
Delaware	5 (2.0)	174 (1.6)	2.9	10 (4.0)	294 (2.7)	3.4	45 (18.1)	814 (7.5)	5.5	55 (22.2)	1,108 (10.2)	5.0
District of Columbia	8 (1.9)	213 (2.2)	3.8	14 (3.4)	354 (3.7)	4.0	48 (11.6)	662 (6.9)	7.3	62 (15.0)	1,016 (10.6)	6.1
Florida	149 (4.5)	3,926 (1.8)	3.8	288 (8.8)	6,606 (3.0)	4.4	730 (22.2)	16,245 (7.3)	4.5	1,018 (31.0)	22,851 (10.2)	4.5
Georgia	88 (5.0)	2,633 (2.0)	3.3	175 (9.9)	4,243 (3.3)	4.1	375 (21.3)	10,513 (8.1)	3.6	550 (31.3)	14,756 (11.4)	3.7
Hawaii	20 (4.4)	265 (1.5)	7.5	47 (10.2)	475 (2.7)	9.9	104 (22.7)	1,354 (7.7)	7.7	151 (32.9)	1,829 (10.4)	8.3
Idaho	12 (4.5)	275 (1.2)	4.4	27 (10.2)	508 (2.3)	5.3	61 (23.0)	1,433 (6.5)	4.3	88 (33.2)	1,941 (8.8)	4.5
Illinois	186 (4.2)	2,476 (1.7)	7.5	382 (8.6)	4,367 (2.9)	8.7	768 (17.3)	11,184 (7.5)	6.9	1,150 (25.9)	15,551 (10.4)	7.4
Indiana	34 (4.3)	1,285 (1.6)	2.6	85 (10.7)	2,264 (2.8)	3.8	167 (21.0)	5,823 (7.1)	2.9	252 (31.7)	8,087 (9.8)	3.1
Iowa	21 (3.3)	487 (1.3)	4.3	46 (7.2)	898 (2.3)	5.1	121 (18.9)	2,626 (6.8)	4.6	167 (26.1)	3,524 (9.2)	4.7
Kansas	29 (6.4)	583 (1.6)	5.0	49 (10.8)	974 (2.7)	5.0	89 (19.6)	2,521 (6.9)	3.5	138 (30.5)	3,495 (9.6)	3.9
Kentucky	23 (4.4)	941 (1.7)	2.4	41 (7.8)	1,683 (3.1)	2.4	104 (19.7)	4,409 (8.1)	2.4	145 (27.5)	6,092 (11.1)	2.4
Louisiana	47 (7.6)	1,280 (2.1)	3.7	81 (13.1)	2,142 (3.5)	3.8	170 (27.4)	5,583 (9.1)	3.0	251 (40.5)	7,725 (12.7)	3.2
Maine	9 (4.2)	164 (1.3)	5.5	17 (7.9)	286 (2.3)	5.9	38 (17.7)	780 (6.3)	4.9	55 (25.6)	1,066 (8.7)	5.2
Maryland	110 (5.0)	1,398 (2.0)	7.9	180 (8.2)	2,278 (3.2)	7.9	352 (16.1)	5,213 (7.3)	6.8	532 (24.3)	7,491 (10.5)	7.1
Massachusetts	94 (2.7)	886 (1.3)	10.6	185 (5.2)	1,592 (2.3)	11.6	545 (15.4)	4,680 (6.6)	11.6	730 (20.6)	6,272 (8.9)	11.6
Michigan	85 (5.0)	1,903 (1.7)	4.5	180 (10.7)	3,316 (3.0)	5.4	369 (21.9)	8,090 (7.3)	4.6	549 (32.6)	11,406 (10.2)	4.8
Minnesota	62 (4.5)	898 (1.3)	6.9	116 (8.4)	1,630 (2.4)	7.1	259 (18.8)	4,481 (6.5)	5.8	375 (27.2)	6,111 (8.9)	6.1
Mississippi	10 (4.2)	877 (2.3)	1.1	16 (6.8)	1,451 (3.9)	1.1	75 (31.6)	3,610 (9.7)	2.1	91 (38.4)	5,061 (13.5)	1.8
Missouri	58 (5.7)	1,213 (1.7)	4.8	109 (10.7)	2,131 (2.9)	5.1	236 (23.3)	5,571 (7.6)	4.2	345 (34.0)	7,702 (10.5)	4.5
Montana	8 (5.3)	152 (1.3)	5.3	14 (9.2)	261 (2.2)	5.4	40 (26.3)	857 (7.3)	4.7	54 (35.5)	1,118 (9.5)	4.8
Nebraska	22 (6.0)	348 (1.3)	6.3	40 (10.8)	677 (2.6)	5.9	101 (27.4)	1,879 (7.3)	5.4	141 (38.2)	2,556 (9.9)	5.5
Nevada	22 (3.8)	606 (1.7)	3.6	49 (8.6)	1,052 (2.9)	4.7	136 (23.7)	2,781 (7.8)	4.9	185 (32.3)	3,833 (10.7)	4.8
New Hampshire	6 (2.0)	148 (1.2)	4.1	12 (3.9)	266 (2.2)	4.5	56 (18.3)	744 (6.1)	7.5	68 (22.2)	1,010 (8.3)	6.7
New Jersey	152 (3.7)	1,501 (1.5)	10.1	297 (7.1)	2,671 (2.6)	11.1	708 (17.0)	6,942 (6.9)	10.2	1,005 (24.1)	9,613 (9.5)	10.5
New Mexico	21 (13.6)	377 (1.6)	5.6	29 (18.8)	653 (2.7)	4.4	29 (18.8)	1,782 (7.5)	1.6	58 (37.7)	2,435 (10.2)	2.4
New York	274 (3.7)	3,415 (1.5)	8.0	565 (7.6)	5,938 (2.6)	9.5	1,220 (16.4)	14,669 (6.4)	8.3	1,785 (23.9)	20,607 (9.0)	8.7
North Carolina	110 (5.8)	2,338 (1.9)	4.7	217 (11.5)	3,851 (3.2)	5.6	379 (20.1)	8,740 (7.3)	4.3	596 (31.6)	12,591 (10.5)	4.7
North Dakota	13 (8.4)	151 (1.4)	8.6	19 (12.3)	265 (2.5)	7.2	39 (25.3)	679 (6.3)	5.7	58 (37.7)	944 (8.8)	6.1
Ohio	72 (4.0)	2,449 (1.8)	2.9	143 (7.9)	4,098 (3.0)	3.5	380 (20.9)	10,070 (7.4)	3.8	523 (28.7)	14,168 (10.4)	3.7
Oklahoma	20 (5.1)	870 (1.7)	2.3	42 (10.8)	1,489 (3.0)	2.8	83 (21.3)	4,103 (8.2)	2.0	125 (32.1)	5,592 (11.1)	2.2
Oregon	26 (3.4)	511 (1.2)	5.1	62 (8.2)	940 (2.2)	6.6	175 (23.1)	2,700 (6.2)	6.5	237 (31.3)	3,640 (8.3)	6.5
Pennsylvania	97 (3.8)	2,299 (1.7)	4.2	207 (8.0)	3,912 (2.8)	5.3	443 (17.1)	9,057 (6.6)	4.9	650 (25.1)	12,969 (9.4)	5.0
Puerto Rico	—^¶^ (—)	421 (1.7)	1.0	9 (10.3)	750 (3.1)	1.2	28 (32.2)	2,033 (8.4)	1.4	37 (42.5)	2,783 (11.4)	1.3
Rhode Island	16 (5.4)	175 (1.6)	9.1	22 (7.5)	288 (2.7)	7.6	44 (14.9)	594 (5.6)	7.4	66 (22.4)	882 (8.3)	7.5
South Carolina	31 (4.7)	1,093 (1.9)	2.8	63 (9.6)	1,897 (3.3)	3.3	154 (23.4)	4,499 (7.9)	3.4	217 (33.0)	6,396 (11.2)	3.4
South Dakota	5 (4.3)	168 (1.4)	3.0	14 (12.2)	280 (2.3)	5.0	17 (14.8)	845 (7.0)	2.0	31 (27.0)	1,125 (9.3)	2.8
Tennessee	27 (3.6)	1,325 (1.6)	2.0	56 (7.5)	2,383 (2.9)	2.3	178 (23.7)	6,579 (8.1)	2.7	234 (31.2)	8,962 (11.1)	2.6
Texas	403 (6.6)	6,395 (1.7)	6.3	728 (11.9)	11,138 (2.9)	6.5	1,447 (23.7)	29,265 (7.7)	4.9	2,175 (35.6)	40,403 (10.6)	5.4
Utah	62 (6.2)	643 (1.3)	9.6	112 (11.2)	1,117 (2.3)	10.0	252 (25.1)	3,471 (7.1)	7.3	364 (36.3)	4,588 (9.4)	7.9
Vermont	— (—)	70 (1.2)	5.7	7 (5.3)	111 (2.0)	6.3	18 (13.6)	314 (5.6)	5.7	25 (18.9)	425 (7.5)	5.9
Virginia	96 (4.5)	1,660 (1.7)	5.8	168 (7.9)	2,762 (2.8)	6.1	356 (16.7)	6,820 (6.8)	5.2	524 (24.6)	9,582 (9.5)	5.5
Washington	36 (2.3)	982 (1.1)	3.7	100 (6.3)	1,878 (2.1)	5.3	242 (15.2)	5,456 (6.2)	4.4	342 (21.5)	7,334 (8.4)	4.7
West Virginia	— (—)	343 (1.8)	0.9	16 (11.4)	573 (3.1)	2.8	35 (25.0)	1,664 (8.9)	2.1	51 (36.4)	2,237 (12.0)	2.3
Wisconsin	49 (5.2)	938 (1.4)	5.2	104 (11.0)	1,699 (2.6)	6.1	179 (19.0)	4,561 (7.0)	3.9	283 (30.0)	6,260 (9.6)	4.5
Wyoming	8 (10.5)	81 (1.2)	9.9	10 (13.2)	144 (2.1)	6.9	9 (11.8)	472 (6.8)	1.9	19 (25.0)	616 (8.9)	3.1
**Total**	**3,282 (4.5)**	**61,810 (1.6)**	**5.3**	**6,393 (8.7)**	**107,167 (2.8)**	**6.0**	**14,119 (19.2)**	**278,342 (7.2)**	**5.1**	**20,512 (27.8)**	**385,509 (9.9)**	**5.3**

**Abbreviations:** ART = assisted reproductive technology; PTB = preterm birth; VPTB = very preterm birth.

* ART totals include infants conceived from ART procedures performed in 2016 and born in 2017 and infants conceived from ART procedures performed in 2017 and born in 2017. Total ART births exclude births to non-U.S. residents and include only infants with gestational age data available.

^†^ In cases of missing residency data (0.3%), the patient’s residence was assigned as the location where the ART procedure was performed.

^§^ U.S. births include births to non-U.S. residents (**Source:** CDC Wonder [Internet]. Natality public use data 2007–2017. Atlanta, GA: US Department of Health and Human Services, CDC; 2018).

^¶^ To protect confidentiality, cells with values of 1–4 for ART infants and cells with values of 0–9 for all infants are suppressed. Also suppressed are data that can be used to derive suppressed cell values. These values are included in the totals.

Among singletons only, the percentage of infants who had low birthweight was 8.1% among ART-conceived infants and 6.6% among all infants. In addition, among singletons, the percentage of infants who were born preterm was 14.0% among ART-conceived infants and 8.1% among all infants, and the percentage of SGA infants was 7.6% among ART-conceived infants and 9.9% for infants with gestational age of 22–44 weeks (Table 7).

**TABLE 7:** Percentage of low birthweight (<2,500 g), preterm (<37 weeks), and small for gestational age infants among singleton infants born with assisted reproductive technology* and all U.S. infants, by female patient’s reporting area of residence^†^ at time of treatment — United States and Puerto Rico, 2017

Patient’s reporting area of residence	Low birthweight (<2,500 g)		Preterm (<37 wks)		Small for gestational age (22–44 wks)	
	ART infants (%)	All infants (%)	ART infants (%)	All infants (%)	ART infants (%)	All infants (%)
Alabama	11.6	8.1	23.0	9.9	8.8	11.4
Alaska	9.8	4.8	11.5	7.4	10.0	6.5
Arizona	8.0	6.0	14.5	7.6	7.0	9.1
Arkansas	8.8	7.5	17.3	9.5	6.8	10.7
California	7.5	5.4	12.3	7.1	8.2	9.3
Colorado	9.1	7.3	16.1	7.1	10.4	13.0
Connecticut	7.8	6.1	13.2	7.5	7.1	9.7
Delaware	9.7	7.2	16.5	8.3	8.8	10.2
District of Columbia	6.8	8.5	9.1	8.7	8.2	12.5
Florida	8.7	7.2	14.9	8.5	8.2	10.7
Georgia	7.4	8.0	15.6	9.4	5.9	11.9
Hawaii	8.2	6.8	14.3	8.7	9.4	10.8
Idaho	9.9	5.3	15.2	6.9	6.3	8.6
Illinois	8.2	6.6	13.5	8.4	7.7	9.6
Indiana	7.5	6.6	13.4	8.0	5.5	9.5
Iowa	8.0	5.1	14.3	7.4	6.6	7.2
Kansas	5.5	5.7	13.5	7.8	4.2	8.2
Kentucky	6.9	7.0	13.7	9.2	5.2	9.9
Louisiana	10.4	8.7	19.5	10.6	6.9	11.2
Maine	8.2	5.7	12.5	7.2	7.0	8.7
Maryland	9.2	7.1	15.3	8.6	7.5	9.9
Massachusetts	7.3	5.7	12.3	7.0	7.9	9.4
Michigan	7.8	6.9	14.4	8.2	5.8	10.0
Minnesota	7.4	5.2	14.3	7.1	6.7	7.8
Mississippi	6.1	9.6	20.2	11.4	5.5	13.0
Missouri	8.1	6.9	14.7	8.5	6.3	9.4
Montana	8.3	6.3	20.8	7.8	6.3	9.7
Nebraska	5.8	5.6	18.4	7.8	2.5	7.7
Nevada	7.6	7.5	16.8	8.9	7.7	11.4
New Hampshire	7.3	5.3	12.1	6.6	10.2	8.5
New Jersey	8.5	6.1	14.7	7.6	8.1	9.9
New Mexico	19.6	8.0	17.2	8.8	16.0	12.7
New York	8.1	6.3	12.7	7.2	8.6	10.4
North Carolina	7.8	7.6	14.1	8.6	7.6	10.9
North Dakota	—^¶^	4.9	14.3	6.9	6.0	7.5
Ohio	7.6	6.9	12.7	8.5	7.0	9.9
Oklahoma	7.8	6.6	13.1	9.4	5.3	8.9
Oregon	7.9	5.2	14.6	6.7	5.9	8.1
Pennsylvania	8.0	6.7	13.3	7.6	6.6	10.0
Puerto Rico	—	9.3	14.9	10.3	—	14.5
Rhode Island	6.8	5.7	10.2	6.5	7.7	9.3
South Carolina	7.4	7.8	16.2	9.2	5.6	10.7
South Dakota	—	5.0	8.2	7.3	—	7.3
Tennessee	8.0	7.5	19.8	9.3	7.3	10.4
Texas	9.4	6.7	17.3	8.8	7.2	9.9
Utah	9.7	5.4	17.2	7.5	8.2	8.8
Vermont	6.5	5.1	11.7	6.0	7.5	9.1
Virginia	8.1	6.6	13.1	7.7	7.8	10.1
Washington	7.5	5.2	10.8	6.8	7.8	8.2
West Virginia	11.1	7.7	23.7	10.1	—	10.5
Wisconsin	5.9	6.0	13.4	7.8	4.8	8.5
Wyoming	—	7.3	7.8	—	—	12.4
**Total**	**8.1**	**6.6**	**14.0**	**8.1**	**7.6**	**9.9**

**Abbreviation:** ART = assisted reproductive technology.

* ART totals include infants conceived from ART procedures performed in 2016 and born in 2017 and infants conceived from ART procedures performed in 2017 and born in 2017. Total ART births exclude births to non- U.S. residents and include only infants with gestational age data available.

^†^ In cases of missing residency data (0.3%), the patient’s residence was assigned as the location where the ART procedure was performed.

^§^ U.S. births include births to non-U.S. residents (**Source:** CDC Wonder [Internet]. Natality public use data 2007–2017. Atlanta, GA: US Department of Health and Human Services, CDC; 2018).

^¶^ To protect confidentiality, cells with values of 1–4 for ART infants and cells with values of 0–9 for all infants are suppressed. Also suppressed are data that can be used to derive suppressed cell values. These values are included in the totals.

## Discussion

### Overview

The use of ART has increased substantially in the United States since the beginning of ART surveillance. In 1996 (the first full year for which ART data were reported to CDC), 20,597 infants were born from 64,036 ART procedures performed by 302 reporting clinics. Since then, the number of procedures reported and the number of infants born from ART procedures have more than tripled, and the number of clinics performing ART services has also increased substantially. Multiple improvements can be observed in ART outcomes by comparing years 2016 (*27*) and 2017. The percentage of singleton births increased from 68.5% to 73.6% (a 7.4% increase), and the percentage of twin births decreased from 30.4% to 25.5% (a 16.1% decrease). The percentage of triplets and higher-order births decreased from 1.1% to 0.9%. The percentage of low birthweight among ART-conceived infants decreased from 23.6% to 20.2%, and preterm birth rates among ART-conceived infants decreased from 29.9% to 27.8%. The contribution of ART-conceived twins to all twins born in the United States decreased from 16.2% to 14.7%. The contribution of ART-conceived infants to all triplets and higher-order infants decreased from 19.4% to 17.3%.

Despite these declines, ART disproportionally contributes to multiple births and poor birth outcomes (low birthweight and preterm birth). In 2017, the multiple-birth rate was nearly eight times higher among ART-conceived infants compared with all infants (26.4% versus 3.4%). Although infants conceived with ART accounted for approximately 1.9% of total births in the United States, the proportion of multiple births attributable to ART was 14.7%. The percentage of infants with low birthweight or born preterm was 2.4 and 2.8 times higher among ART-conceived infants (20.2% and 27.8%, respectively) than among all infants (8.3% and 9.9%, respectively). Nationally, even among singletons, the rate of preterm birth among ART-conceived infants was two times the preterm birth rate among all infants. Because ART infants are more likely to be multiple births than infants among the general population, their contribution to adverse outcomes such as preterm birth continues to be noteworthy.

### Variations in ART Use by Reporting Area 

The rate of ART use, as measured by number of procedures performed per 1 million women of reproductive age, declined slightly from 3,075 to 3,040 between reporting years 2016 and 2017. ART use varied across areas. Residents of 14 states (Connecticut, Delaware, District of Columbia, Hawaii, Illinois, Maryland, Massachusetts, New Hampshire, New Jersey, New York, Rhode Island, Utah, Vermont, and Virginia) had higher rates of ART use than the national rate.

Residents of California, Illinois, Massachusetts, New Jersey, New York, and Texas accounted for almost half (48.2%) of all infants conceived with ART. The large number of ART procedures performed in these six states is a result of both the size of the general population (e.g., California and Texas) and high rates of ART use per capita (e.g., Massachusetts, Illinois, New Jersey, and New York).

The contribution of ART to all infants born varied substantially by state. State-level differences might be explained in part by variations in health insurance coverage and disparities in access to fertility services. Seventeen states (Arkansas, California, Connecticut, Delaware, Hawaii, Illinois, Louisiana, Maryland, Massachusetts, Montana, New Hampshire, New Jersey, New York, Ohio, Rhode Island, Texas, and West Virginia) have laws mandating that private insurers provide coverage for some fertility treatments, although not all mandates require coverage for ART (*28*,*29*). Eleven states (Arkansas, Connecticut, Delaware, Hawaii, Illinois, Maryland, Massachusetts, New Hampshire, New Jersey, New York, and Rhode Island) have insurance mandates that cover at least one ART cycle. Six states (California, Louisiana, Montana, Ohio, Texas, and West Virginia) have insurance mandates that exclude IVF coverage. Mandates from four states (Illinois, Massachusetts, New Jersey, and Rhode Island) include comprehensive coverage for at least four oocyte retrievals. In addition, Connecticut also covers up to two IVF cycles, with a maximum of two embryos transferred. Three of the four states with comprehensive mandates (Illinois, Massachusetts, and New Jersey) had rates of ART use that were at least 50% higher than the national rate. Insurance mandates for infertility treatments have been associated with greater use of ART (*30*–*32*). Other possible contributors to differences in ART use across states might include factors affecting access to fertility services, such as infertility diagnosis, evaluation, and treatment (*33*).

Race and ethnicity might be associated with use of fertility services. One study that analyzed 2014 NASS data showed that ART use was highest among Asians/Pacific Islanders, followed by White non-Hispanic women, whereas non-Hispanic Black, Hispanic, and American Indian/Alaska Native non-Hispanic women had less than the national average levels of use (*33*). Because many insurance plans in the United States do not cover ART treatment, costs are often the responsibility of individual patients (*30*). Even in states with an insurance mandate, ART use rates among women aged 15*–*44 years for non-Hispanic Black and Hispanic women were lower than the overall national use rate (*33*). A study that linked NASS data to state vital registries data in Florida, Massachusetts, and Michigan also found disparities by maternal race and ethnicity in ART use. However, limitations of data collection systems in terms of data quality and completeness of race and ethnicity data may limit meaningful research in racial and ethnic disparities in ART use and outcomes (*33*). As of 2016, all states had adopted the 2003 revision of the birth certificate that includes information on whether the pregnancy resulted from the use of infertility treatment; 47 states and the District of Columbia differentiate between the use of ART and non-ART treatments. Improved data collection and data linkages to obtain accurate data on race and ethnicity would facilitate research in this area. In 2014, *CDC’s National Public Health Action Plan for the Detection, Prevention, and Management of Infertility* identified racial disparities in the prevalence, diagnosis, referral, and treatment of infertility as a public health priority and called for improved monitoring of infertility treatment services to reduce disparities in infertility treatments and outcomes (*34*).

### Single-Embryo Transfer Rates

Recommendations issued by the American Society for Reproductive Medicine and SART to limit the number of embryos transferred have been revised multiple times to reduce higher-order multiple deliveries (*25*,*35*–*37*). However, the most recent guidance in 2017 also was aimed at reducing all multiple births, including twins (*5*). Because of the change in clinical practice, in 2017, NASS surveillance began reporting SET rates among all embryo transfers instead of eSET for patients using fresh embryos from their own fresh eggs, which is different from previous reports (*1**,**25*). Although this transition allows a more accurate representation of current embryo-transfer practices, it limits the assessment of changes in these practices over time using data from previous reports. Similar to variations by state in eSET, variations in the percentage of SET procedures across states and territories suggest that SET might not be implemented equally in all areas.

### ART Multiple Births 

Singleton births have lower risks than multiple births for adverse birth outcomes, such as prematurity, low birthweight, developmental disability, and death (*13*,*38*,*39*). To optimize healthy birth outcomes, the transfer of fewer embryos should be encouraged when clinically appropriate, taking into consideration the patient’s age and prognosis (*5*,*40*). The percentage of multiple births among ART-conceived infants in the United States decreased from 53.1% in 2000 (when national multiple birth rates were first reported in the *Surveillance Summary*) to 26.4% in 2017 (*41*). A substantial decrease was noted for both the percentage of ART-conceived triplets and higher-order infants (from 8.9% in 2000 to 0.9% in 2017) and the percentage of ART-conceived twins (from 44.2% in 2000 to 25.5% in 2017). States with the highest SET rates (i.e., Delaware, the District of Columbia, and Massachusetts) also had the lowest rates of ART-conceived multiple births.

Transferring two embryos is associated with a slight increase in the overall birth rate but a greater increase in the twin birth rate compared with transferring a single embryo (*42*,*43*). However, transferring two embryos sequentially (single-embryo transfer over two sequential procedures, if the first procedure did not result in live birth) has similar cumulative live-birth rates and substantially lower twin delivery rates than transferring two embryos in a single procedure and might be a cost-effective approach, in which estimated costs include ART treatment and pregnancy- and infant-associated medical costs (*42*,*43*). Evidence from other countries suggests that access to coverage for ART, availability of cryopreservation services, and economic and social factors regarding the number of embryos transferred per cycle can encourage SET procedures and reduce multiple births (*44*). In 2013, the mean health care costs to patients and insurers were estimated at $26,922 for ART-conceived singleton deliveries, $115,238 for ART-conceived twin deliveries, and $434,668 for ART-conceived triplets and higher-order infants (*45*).

The desire for twins among couples experiencing infertility and their perception that the benefits of a multiple-gestation pregnancy (compared with no pregnancy) outweigh the risks (*46*–*48*) might partially explain why twin rates remain high. Therefore, understanding the perspective of couples undergoing infertility treatments regarding multiple-gestation pregnancies and multiple births is important. Patient education focusing on maternal and perinatal morbidity and mortality and the economic costs of twin gestations has been effective in reducing the preference for twins among patients undergoing ART (*49*–*51*).

### ART Low Birthweight Infants, Preterm Births, and Small for Gestational Age Infants

In the United States, although rates of ART-conceived preterm and low birthweight infants have been declining steadily, the percentage of infants born with low birthweight and preterm was higher among ART-conceived infants (20.2% and 27.8%, respectively) than among all infants (8.3% and 9.9%, respectively). In addition, among ART-conceived infants, preterm and low birthweight rates varied substantially across states and territories. For example, the percentage of ART-conceived infants born in gestational weeks 34–36 varied from 11.6% (in the District of Columbia) to 32.2% (in Puerto Rico), whereas less variation by state was observed for same gestational week (34–36 weeks) category among all infants (range: 5.6% in Vermont to 9.7% in Mississippi).

Fertility treatments, both ART and non-ART, contribute substantially to preterm births (*38*,*52*). Preterm births are a leading cause of infant morbidity and mortality, and preterm infants are at higher risk for health and developmental problems and death than full-term infants (*38*,*53*–*55*). The health risks associated with preterm birth have contributed to increased health care costs. In 2016, the societal economic cost associated with all preterm births in the United States was estimated at $25.2 billion annually ($64,815 per infant born preterm) based on updates to estimates originally reported by a 2007 Institute of Medicine report (*38*) (https://www.marchofdimes.org/peristats/documents/Cost_of_Prematurity_2019.pdf). The societal economic cost associated with ART-conceived preterm infants in the United States in 2017 was also re-estimated at approximately $1.3 billion on the basis of estimates computed in 2012 (*56*). Furthermore, the economic costs of multiple births underscore the importance of efforts to reduce ART-related multiple births, which in turn would reduce preterm births. 

In addition to the known risks for multiple births associated with ART, singleton infants conceived with ART procedures are at increased risk for preterm birth (14.0%) compared with all singleton infants (8.1%). Among singletons, SGA rates were lower among ART-conceived infants compared with all infants. Other studies have shown variability in SGA risk by fresh versus frozen and donor versus autologous cycles (*57*,*58*). Although low birthweight is a risk factor for adverse effects among newborns and is usually associated with preterm births, SGA might be a better indicator of these risk factors among newborns because it accounts for gestational age (*57*). More research is needed to better understand the risk for SGA among ART-conceived infants and how risk for SGA might vary by the type of ART cycle performed.

Use of ART only partially explains the overall prevalence of adverse outcomes such as multiple births in the United States. Other factors influencing multiple births include advanced maternal age at conception and the use of non-ART fertility treatments (*38*,*52*,*59*,*60*). During 1980–2009, a substantial increase in the number of twin infants occurred due to the older age of women giving birth (*59*). The risk for multiple gestations associated with non-ART fertility treatments (i.e., controlled ovarian stimulation and ovulation induction coupled with timed intercourse or intrauterine insemination) is less well-documented than that associated with ART procedures; fertility clinics are only required to report data on ART use to NASS. However, research suggests that non-ART fertility treatments contribute to a larger percentage of multiple births than ART. In 2015, approximately 17% of multiple births in the United States were attributable to IVF fertility treatments, whereas 29% were attributable to non-IVF fertility treatments (*61*,*62*).

Additional efforts are needed to monitor the use of non-ART fertility treatments and their role in multiple births, particularly because the ability to control the occurrence of a multiple birth is more challenging when using non-ART fertility treatments (*52*). CDC is monitoring the prevalence of ART and non-ART fertility treatment use and resultant outcomes among women who had live births in certain states participating in the Pregnancy Risk Assessment Monitoring System (*63*,*64*). 

## Limitations

The findings in this report are subject to several limitations. First, ART surveillance data were reported for each ART procedure performed rather than for each patient who used ART. As a result, because patients can undergo multiple procedures, measures of ART use are an approximation; certain women who use ART are younger or older than the age range of 15–44 years, and certain women might have had more than one procedure during the reporting period. Therefore, the procedure-specific use rates reported here might be higher than the actual per-patient use rates. Second, when comparisons are made between ART-conceived births and all births, all births include the ART-related births. Similarly, when comparing outcomes for ART-conceived infants versus all infants, the denominator for all infants born includes ART-conceived infants. Third, preterm birth, low birthweight, and being small for gestational age could be associated with factors contributing to underlying infertility or other maternal or paternal factors and not necessarily ART procedures. Fourth, approximately 10% of fertility clinics that performed ART in 2017 did not report their data to CDC. Although these clinics might have had results differing from reporting clinics, typically, they are smaller and represent approximately 2% of all ART cycles performed in the United States (*1*). Fifth, SET rates cannot be compared with eSET rates from previous years because of differences in definition between eSET and SET rates. In addition, in previous reports, eSET rates were reported only for procedures in which patients were using their own fresh eggs. SET rates were calculated for all procedures except procedures in which banking was performed for future ART use (and therefore no eggs or embryos were retrieved) and procedures that were considered research cycles. Sixth, comparisons between ART births and all U.S. births should be limited because some ART calculations might exclude births to non-U.S. residents because the data are reported by mother’s state of residence. However, the NVSS data that are derived from the birth certificates include all births, including births to non-U.S. residents. Seventh, gestational age is computed for ART infants conceived with frozen embryos by subtracting 17 days (to allow for an average of 3 days in embryo culture) from the date of transfer. However, many frozen-embryo transfers use blastocyst embryos (approximately 5 days of embryo culture), which might slightly underestimate gestational age. Finally, the number of ART procedures reported for 2017 included all procedures in which banking was not performed, including procedures with frozen eggs that were thawed, and therefore comparisons with data from previous years (2015 and earlier) in which procedures using thawed eggs were excluded from analyses should be made with caution. 

## Conclusion

Since 1995, the number of ART procedures performed in the United States and the number of infants born as a result of ART procedures have more than tripled. With this increasing use, ART-conceived infants represented approximately 2% of infants born in the United States in 2017 and contributed substantially to the prevalence of low birthweight and preterm births. Furthermore, among ART-conceived infants, although the percentage of all multiple births has decreased since 2000, the percentage of twins, which has also declined, still remains high (26%). Because of higher rates of preterm birth among multiple births, ART has a disproportionate number of poor birth outcomes. This report provides information that allows state health departments working with patients and clinical organizations to monitor the extent of ART-related adverse perinatal outcomes in their regions and take action to initiate programs and policies to reduce the adverse effects of ART multiple births. A state-specific website that presents selected ART success rates and other statistics is available (https://www.cdc.gov/art/state-specific-surveillance/index.html).
